# Parenting and climate change: assessing carbon capability in early parenthood

**DOI:** 10.1007/s11111-025-00506-6

**Published:** 2025-09-25

**Authors:** Sam Hampton, Elodie Taylor, Lorraine Whitmarsh

**Affiliations:** Department of Psychology, University of Bath, 10 West, Claverton Down, Bath, BA2 7AY UK

**Keywords:** Carbon capability, Parenting, Parenthood, Children, Climate change

## Abstract

Climate change is an intergenerational issue, with parents uniquely positioned to influence both current emissions and future generations’ environmental attitudes. This study explores the attitudes, beliefs, and behaviours of parents in the UK regarding climate change, assessing their ‘carbon capability’. Using data from a nationally representative survey (*n* = 1001), in-depth interviews (*n* = 30), and focus groups (*n* = 7), we found that parenthood is associated with increased energy consumption, transport use, and plastic waste. Despite these challenges, parents demonstrated a high capacity to influence and be influenced by others. They were aware of their environmental impacts and were open to adopting pro-environmental behaviours, driven by a desire to prepare and protect their children. Parents preferred timely information provision as a policy response but recognise the need for more substantive, structural interventions to support sustainable living. This study highlights the critical role of parents in climate action and calls for targeted policies to enhance their carbon capability. By applying the carbon capability framework, which integrates individual and structural factors, this research contributes to both parenting and environmental psychology literatures. Our findings underscore the importance of empowering parents with the knowledge and tools necessary to reduce their carbon footprints and foster a new generation of climate-conscious citizens.

**Supplementary information:**

The online version contains supplementary material available at 10.1007/s11111-025-00506-6.

## Introduction

Climate change is an inherently intergenerational issue, where future generations shoulder the greatest costs (IPCC, 2022). In this context, parents hold a unique position, bearing a degree of responsibility for past and current emissions, alongside deep concern for their children’s wellbeing and prospects. Looking simultaneously into the past and the future, parents occupy a pivotal role in addressing climate change, as their decisions and actions today can significantly influence future outcomes.

Becoming a parent is one of the most significant moments of change in the lifecourse, marked by disrupted routines, recalibrated priorities, and opportunities for forming new behaviours (Thompson et al., 2011). These transitions provide a critical window for fostering parents’ carbon capability—defined as “the ability to make informed judgments and to take effective decisions regarding the use and management of carbon, through both individual behaviour change and collective action” (Seyfang et al., 2007). However, the literature presents a complex picture: while many parents report high levels of concern about climate change (Hp, 2023) and experience heightened climate anxiety (Blum, 2020), the link between parenthood and sustained pro-environmental behaviour is far from straightforward. For instance, despite their concern, some parents adopt distancing strategies to manage emotional distress, whereas others embrace parenthood as an opportunity for moral action and personal growth (Bechard et al., 2023). Moreover, one quantitative study published in this journal found no evidence that having children leads to pro-environmental behaviour change (Thomas et al., 2018). This finding raises important questions about why parenting transitions do not consistently translate into environmental action and what can be done to support parents in leveraging these moments of change.

Research highlights that parents are deeply concerned about climate change: one study involving 5000 parents from five countries found that 91% worry about the climate crisis (Hp, 2023). This concern not only often translates into heightened climate anxiety (Blum, 2020) but also creates opportunities for action. A systematic review found that while some parents use distancing strategies to cope with emotional distress about future climate impacts, others embrace these challenges as catalysts for personal growth and morally grounded parenting practices aimed at reducing environmental impacts (Bechard et al., 2023). These findings underscore both barriers and opportunities for enhancing parents’ carbon capability during key moments of transition in family life.

Several studies call for more research on how parenthood impacts climate engagement and how this major life transition can be harnessed for pro-environmental behaviour change (Cripps, 2017; Lawson et al., 2019). This article addresses this gap by applying the concept of carbon capability to parents for the first time. Unlike narrower concepts such as carbon literacy, carbon capability highlights not only individual knowledge, skills, and motivations but also engagement with broader systems of provision and governance. It emphasises that individuals can adopt multiple roles beyond acting as consumers, including as citizens and role models (Hampton & Whitmarsh, 2023, 2024).

Parenting is an important *moment of change*. Moments of change (MoC) refer to significant life transitions—such as becoming a parent—that disrupt routines and create opportunities for new behaviours to emerge (Thompson et al., 2011). These transitions are particularly relevant for enhancing carbon capability, as they offer critical windows for fostering new environmental practices. This study focuses on parents of children aged under 15 because this group is likely to experience ongoing lifestyle changes related to raising children, including shifts in consumption patterns, housing needs, and transportation habits. Despite its strengths, the carbon capability framework has not yet been applied to parents or MoC (Burningham & Venn, 2020). This article seeks to reinvigorate carbon capability by developing a new framework focused on parents and applying it to empirical data. Drawing on a nationally representative survey of UK residents (*n* = 1001), as well as in-depth interviews (*n* = 30) and focus groups (*n* = 7) with parents of children under 15 in England, this study examines parents’ carbon capabilities compared with those of the wider population. Specifically, the study is guided by two research questions: (1) To what extent are parents in the UK ‘carbon capable’? and (2) What can be done to enhance the carbon capability of parents?

## Literature review

### Parenthood and climate change

Parenthood and climate change intersect at profound ethical crossroads, presenting complex moral responsibilities. Scholars have argued that parents have a unique safeguarding duty with respect to the environment, which is not limited to their own children and grandchildren, but extends to future generations in their entirety (Cripps, 2017; Howard et al., 2021; Sanson et al., 2018). This intergenerational responsibility aligns with concepts of generativity—the psychosocial drive to nurture future generations (Erikson, 1963; McAdams 2004)—and legacy thinking, where parents seek to leave an improved world for their children (Markowitz et al., 2012; Syropoulos & Markowitz 2024). Recent work suggests parenthood heightens these motivations, potentially driving climate action through stewardship and moral responsibility (Shrum et al., 2023; Zaval et al., 2015).

Parents are also responsible for instilling values, modelling behaviours, and (re)asserting social norms through their children (Nche et al., 2019). For instance, parents’ expectations for their children’s academic progress have significant impact on academic performance (Eccles, 2005; Kaplan et al., 2001). Interventions targeting parents have found significant results on childhood outcomes, such as improving weight-related nutrition to prevent diabetes (Golley et al., 2011) and reducing the risk of substance abuse (Vimpani, 2005). Parent–child dynamics with respect to pro-environmental behaviours have attracted scholarly attention relatively recently (Lawson et al., 2019; Nche et al., 2019; Sanson et al., 2018). This literature has demonstrated that fostering a love for nature can lead to environmentally friendly behaviours in adolescence (Evans et al., 2019; Lawson et al., 2019), while a qualitative study in Belgium found that parents had the strongest influence on children’s energy literacy (Pearce et al., 2020).

Evidence of reverse-intergenerational learning—where children influence their parents’ attitudes and behaviours—has been well-documented in the broader parenting literature, spanning topics such as sexuality, technology adoption, and food choices (Baily, 2009; Flurry & Burns, 2005; LaSala, 2000; Pariera & Brody, 2023). This is highly relevant to climate-related behaviours, as it suggests that children can be catalysts for change within the family unit. Although few studies have focused specifically on climate change, some evidence supports this idea: Lawson et al. (2018) found that children who participated in climate change education programmes increased climate concern among their parents, particularly among those initially resistant to such messages. Similarly, Ballantyne et al. (2001) demonstrated that Australian children exposed to environmental education influenced their parents’ attitudes more than their peers who did not receive such instruction. These findings highlight the potential for children to play an active role in shaping family climate engagement and suggest opportunities for policy and educational interventions that target both generations.

Shrum et al. (2023) describe the ‘green parenthood effect’ as a process whereby becoming a parent induces more environmentally friendly behaviours and attitudes. This effect is theorised to emerge from increased legacy focus and generativity, though empirical evidence remains mixed (Alisat et al., 2014; Thomas et al., 2018; Zaval et al., 2015). Recent studies have found that both children and parents can experience significant levels of climate-induced mental distress, with climate anxiety generally found to be higher among women and children than men, and particularly acute among younger people (Bechard et al., 2023; Blum, 2020; Clayton & Karazsia, 2020; Hickman et al., 2021; Thomas et al., 2018). Crucially, climate anxiety is not only a psychological burden but can also motivate some individuals and families to take pro-environmental actions, such as encouraging energy-saving behaviours or buying second-hand items (Whitmarsh et al., 2022), which may foster a sense of empowerment and agency within the household. Supporting parents to enhance their carbon capability—including their knowledge, confidence, and practical skills—could therefore enable families to respond to climate anxiety in more constructive ways, both by adopting pro-environmental behaviours and by modelling coping strategies for their children.

Procreation has itself become a topic of debate and controversy in the context of climate change. On average, carbon emissions from households with one child increase by 25% compared to those without (Nordström et al., 2020). Wynes and Nicholas’s (2017) study attracted global attention when they concluded that having one fewer child was the most effective emission reduction strategy for individuals seeking to take action on climate. Its publication sparked backlash, with opponents citing reproductive freedoms as a human right (Laurence, 2017) and the tendency to place the burden of systemic problems onto individuals and families (Pedersen & Lam, 2018). Nonetheless, voluntary childlessness is on the rise (Schneider-Mayerson & Leong, 2020). While clearly parents are not wholly responsible for climate change, there is widespread agreement that parents have unique opportunity to influence younger generations (Cripps, 2017; Thomas et al., 2018).

Parenting and climate change, however, is an understudied area of research, and a recent review article highlighted key gaps in the literature (Shrum et al., 2023). These include how the transition to parenthood impacts climate engagement, and testing how this MoC might be capitalised upon by campaign groups or policymakers to engage parents on climate related matters. Parenthood features prominently in MoC studies. Thompson et al. (2011) found that car usage decreases following the birth of a child, as new parents stay at home more and shop locally; whereas energy and material consumption increased (e.g. disposable items such as nappies), and food choices altered (including buying healthier but also cheaper foods). Nash et al. (2020) similarly found that reduced finances restrict the ability of new parents to buy healthy products and goods. However, parenthood involves multiple MoCs, from pregnancy onwards. Significant milestones include birth, weaning, parents’ return to work following maternity/paternity leave, the arrival of siblings, and the induction into educational and care environments (nursery, primary, secondary school, university). Each of these can be a marker of change for parents, both in practical ways with direct implications for environmental impacts (energy, transport, and consumption behaviours) and in other ways (values, priorities, identity), which can have equally important, but indirect implications for carbon capability, including citizenship behaviours. Given the wide range of behaviours (e.g. energy, transport, diet, product purchasing), and multiple stages of parenting, as well as the limited literature from which to draw, we have not formulated hypotheses for this study. Instead, we conduct exploratory analyses of a range of domains, informed by our carbon capability framework. To ensure analytic focus and comparability, the study is restricted to parents with children under 15, as this group actively experiences multiple caregiving transitions and is most likely to be making daily decisions that shape family carbon capability.

### Carbon capability

Carbon capability, introduced by Seyfang and colleagues (2007), moves beyond the narrow focus on individual knowledge and climate literacy by policymakers to highlight engagement with structures and systems of provision. They defined carbon capability as “*the ability to make informed judgements and to take effective decisions regarding the use and management of carbon, through both individual behaviour change and collective action*”. Carbon capability also highlights how individuals can adopt multiple roles besides acting as consumers, including being climate citizens and role models (Hampton & Whitmarsh, 2023, 2024). This makes it apt for analysing the role of parents for climate action.

While carbon capability itself has not been applied to parents, there is a substantial literature using the capability approach (Nussbaum, 2000; Sen, 1989, 1999) to understand parent–child relationships. The capability approach provides the theoretical foundation for the concept of carbon capability, bringing attention to the psychological, social, material, and structural factors which influence an individual’s ability to pursue a valued, fulfilling—and sustainable—life (Hampton & Whitmarsh, 2024). It includes three core concepts: functionings, capabilities, and agency. Functionings denote desired states of being, while capabilities represent the ability to achieve given functionings. Agency is the autonomy to pursue valued goals (Comim et al., 2008). Applied to parenting, this approach has been used to investigate how child protection policies impact families (Hartas, 2014) and to illustrate how a child’s functionings *and* capabilities are largely determined by the relationship between a child and his/her parents (Gupta, 2017). Parents are understood to play a major role in shaping their children’s capabilities—including the ability to adopt low-carbon behaviours—as well as influencing the development of values that inform which functionings children pursue, such as ecological responsibility (Gupta, 2017; Hartas, 2014). However, the extent to which parents directly instil ecological values remains an open question, warranting further empirical investigation.

Adopting the framework to analyse the capabilities of urban residents in eastern China, Wei et al. (2016) developed an alternative model for tracing five ‘stages’ of carbon capability. This starts with foundational *carbon values* which drive an individual’s low carbon choices. These are built on with the ability to *identify* options (gaining knowledge), and then to make pro-environmental *choices*. Sustained behaviours amount to carbon ‘*action* capability’, and the final stage is the ability to *influence* others. Most recently, Hampton and Whitmarsh (2024) incorporate this influence capability in their carbon capability model, highlighting also its temporal dimension and the need to distinguish between different roles and actors.

**Fig. 1:**
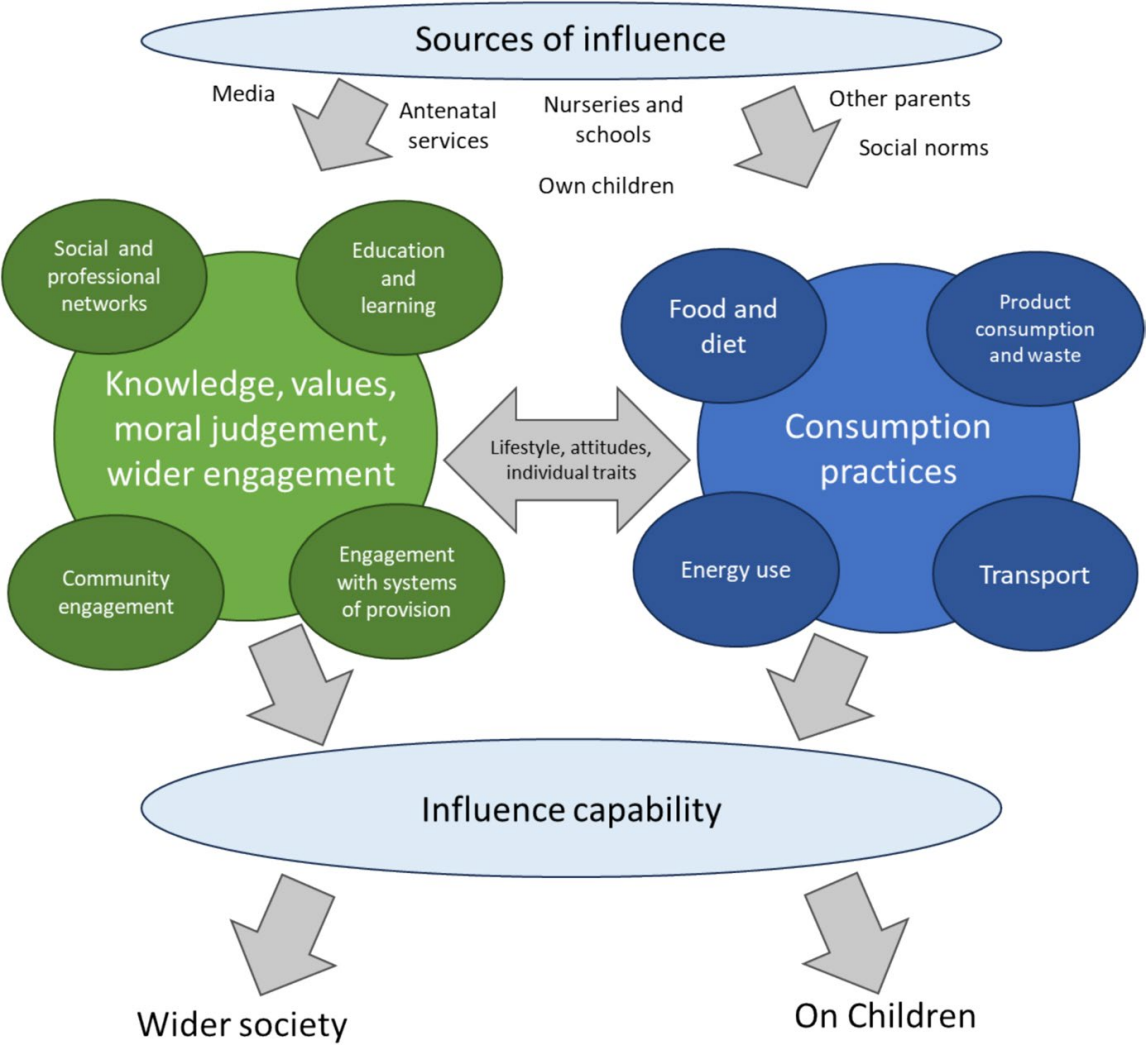
Carbon capability framework for early parenthood

These models each have limitations when applied to parents. While Whitmarsh et al.’s (2011) model accounts for the effect of exogenous factors, it downplays the role of influence and social networks (a point made by Hampton & Whitmarsh, 2024). Wei et al. foreground influence, but this is one way: *on* others. They also emphasise values, but conceive of these as a prerequisite for carbon capability. We instead build on the wider parenting literature to emphasise the dynamic nature of influence, and values and moral judgement as emergent and mutable. Figure 1 depicts our carbon capability model, tailored for parents, which emphasises multiple modes and directions of influence, the family unit, and the importance of values and moral judgement.

In summary, carbon capability has potential as a valuable theoretical framework for investigating parenthood and climate change. Unlike models of behaviour focused exclusively on the individual, it emphasises how everyday practices are shaped by social norms and systems of provision, and it highlights the multiple roles that parents play as consumers, citizens, educators, and role models.

## Methodology

### Study context

This research was conducted in the UK, where demographic and policy trends shape the context for parenting and climate action. The UK has seen a steady decline in birth rates, with the average age of first-time parents rising to 30.9 years for mothers and 33.7 years for fathers (ONS, 2023a, 2023b). Parents are increasingly affected by the cost-of-living crisis and high childcare costs, which influence family planning and daily practices. While the UK government has expanded subsidised childcare for working parents in recent years, broader parenting policies remain focused on child welfare, education, and safeguarding, with little direct emphasis on pro-environmental behaviours.

Across the OECD, information campaigns are a popular instrument for influencing public behaviour, particularly in areas such as health and safety. However, to date, there have been no government-backed campaigns in the UK specifically targeting parents to promote pro-environmental behaviours or climate engagement. In this policy vacuum, charitable organisations have begun developing resources and campaigns to support parents in navigating climate change and advocating for action. *Parents for the Future* (n.d.) facilitates ‘climate conversations’ designed for peer-support and makes it easy for parents to lobby politicians for action. *Climate Outreach* has developed guidance for campaigners and policymakers to engage with parents (Corser, 2022), and the UK branch of *Students Organising for Sustainability* (SOS) provides advice to children to educate their parents on climate change. This context underscores the importance of understanding parents’ attitudes and behaviours regarding climate change, and highlights the potential for targeted policy interventions.

### Data collection

A mixed methods approach was adopted to provide a comprehensive understanding of carbon capability in early parenthood, combining the breadth of quantitative survey data with the depth and contextual richness of qualitative interviews and focus groups. This study was designed as an exploratory investigation of carbon capability in early parenthood and was not pre-registered. The initial survey enabled the collection of nationally representative data on knowledge, attitudes, and behaviours, allowing for robust statistical analysis and generalisability. However, surveys alone are limited in their ability to capture the complexity of personal motivations and the nuanced ways in which parents interpret and enact pro-environmental behaviours. To address these limitations, in-depth interviews were conducted to explore individual experiences, values, and the moral and emotional dimensions of parenting in the context of climate change. Focus groups were subsequently employed to facilitate collective discussion, enabling participants to co-construct and critique potential interventions and policy proposals.

Data collection comprised three main stages from April to August 2022. A two-wave survey (*n* = 1001) was followed by semi-structured interviews (*n* = 30) and two focus groups (*n* = 3, *n* = 4). Qualitative data were conducted by one of the authors, a university student with no children. The other authors, who are both parents of young children, helped to develop the interview protocol, with insights from their own experience.

### Phase 1—survey

A survey comprising a total of 340 questions was issued to a nationally representative sample of UK residents (not only parents), based on age, gender, religion, ethnicity, and educational attainment. The survey was administered by Dynata, a research company. The full survey instrument is provided in Appendix 1. The anonymised dataset generated and analysed during the current study is openly available from the University of Bath Data Repository (Hampton & Whitmarsh 2022). Survey questions were designed using the six domains of carbon capability as developed by Hampton and Whitmarsh (2023, 2024), spanning consumption activities (energy, transport, food, shopping) and indirect actions (influence and citizenship), and focused on individuals’ knowledge, attitudes, and behaviours. Respondents were asked about their willingness to change consumption behaviours, their involvement in public sphere activities (community membership and political participation), and the extent to which they support a range of climate policies. The survey was split into two 20-min questionnaires, 2 weeks apart, due to the length of the survey. Wave 1 (*n* = 2139) focused on energy, transport, and policy. Wave 2 was issued to the same cohort, but only 1118 responses were received. The second wave focused on food, spending habits, influence capabilities and social capital. Only responses to the complete survey are analysed for this study.

Attention checks were included in both waves. Respondents failing any of these were removed prior to a representative sample being obtained (139 responses in wave 1 and 114 in wave 2). Further quality assurance included checks for the number of children per household, the number of heating systems, and any ‘straight line’ answers to Likert scale questions. These quality checks resulted in a total of 1001 verified responses, used for analysis. Attrition analysis revealed that those completing both survey waves were more likely to be women, older, and more educated (see Appendix S4). Wave 2 responses were therefore weighted using a rim weighting technique which enables multi-variate adjustments (Sharot 1986).

Respondents were categorised into four groups using responses to a question on the age of household occupants. Four parent groups are used for analysis, distinguishing between ages of children, and those without children in these age ranges.

- Group 1: children aged 0–7 only (*N* = 80)
- Group 2: children aged 8–15 only (*N* = 112)
- Group 3: children aged both 0–7 and 8–15 (*N* = 36)
- Group 4: no children aged under 15 (*N* = 773)

A limitation of the survey design is that only the presence of children aged 0–7 or 8–15 was recorded. Consequently, the comparison group (‘adults without children under 15’) may include parents of older children as well as adults who have never had children. This should be borne in mind when interpreting group differences. Existing longitudinal evidence suggests that parenthood itself does not have a substantial or lasting impact on environmental attitudes or behaviours (Milfont et al., 2020; Thomas et al., 2018). Moreover, the climate emergency has only become a prominent issue in public debate in recent years, meaning that parents of older children may not have experienced the same sense of urgency or opportunity for change during their own parenting transitions. Nevertheless, because our comparison group may include respondents who are parents of older children, we cannot rule out the possibility that some of their responses were influenced by past experiences of parenthood. This potential overlap should be kept in mind when interpreting group-level differences.

### Phase 2—in-depth interviews

Thirty semi-structured interviews with parents (15 males and 15 females with children under 15) were conducted in July and August 2022. Survey respondents were anonymous, so interview participants were recruited separately, and had *not* also completed the survey. Recruitment methods included online advertisements on parent forums and Facebook groups (*n* = 12) as well as in London-based primary schools’ newsletters (*n* = 7). Participants were also recruited through opportunistic sampling outside a leisure centre (*n* = 3) and through snowballing (*n* = 8). Interview participants were balanced by gender, but the sample is not representative of the UK as a whole, with most participants recruited from London and the south-east. Interviews lasted between 45 and 60 min and were conducted one-on-one, either online (*n* = 22) or in-person (*n* = 8).

Interviews covered similar topics to survey questions (behaviours, attitudes etc.), but delved deeper into issues such as moral responsibility, hope and despair, mother and father roles, and how influence between parents and children changes over time and in different contexts. Male and female parents were interviewed separately to allow an understanding of their individual opinions and a comparison of responses. Participant quotations are annotated with codes indicating their gender (F/M) and the ages of their children (e.g. 6–4).

### Phase 3—focus groups

Two focus groups were conducted. The focus groups were spilt by gender to allow participants to feel more at ease when talking about sensitive topics and to observe any differences in attitudes between mothers and fathers. The first comprised four men and the second included three women, and none were also interviewees. Focus groups were not initially part of the research design, but this method was chosen as a way to explore policies which might enhance parents’ carbon capability. Focus groups are an effective method for generating and testing ideas for policies (O. Nyumba et al., 2018), so discussions focussed on interventions, leverage points, and specific policy proposals.

#### Data analysis

Our analyses were guided by the two research questions. Survey data were used primarily to address RQ1: To what extent are parents in the UK ‘carbon capable’? One-way ANOVAs were conducted to examine differences across parent groups on various measures of carbon capability, followed where necessary by logistic regression analyses to test whether these differences remained significant when controlling for socio-demographic factors (gender, age, education).^1^ For all comparisons, effect sizes (*η*2) were calculated to quantify the magnitude of observed differences.

**Fig. 2:**
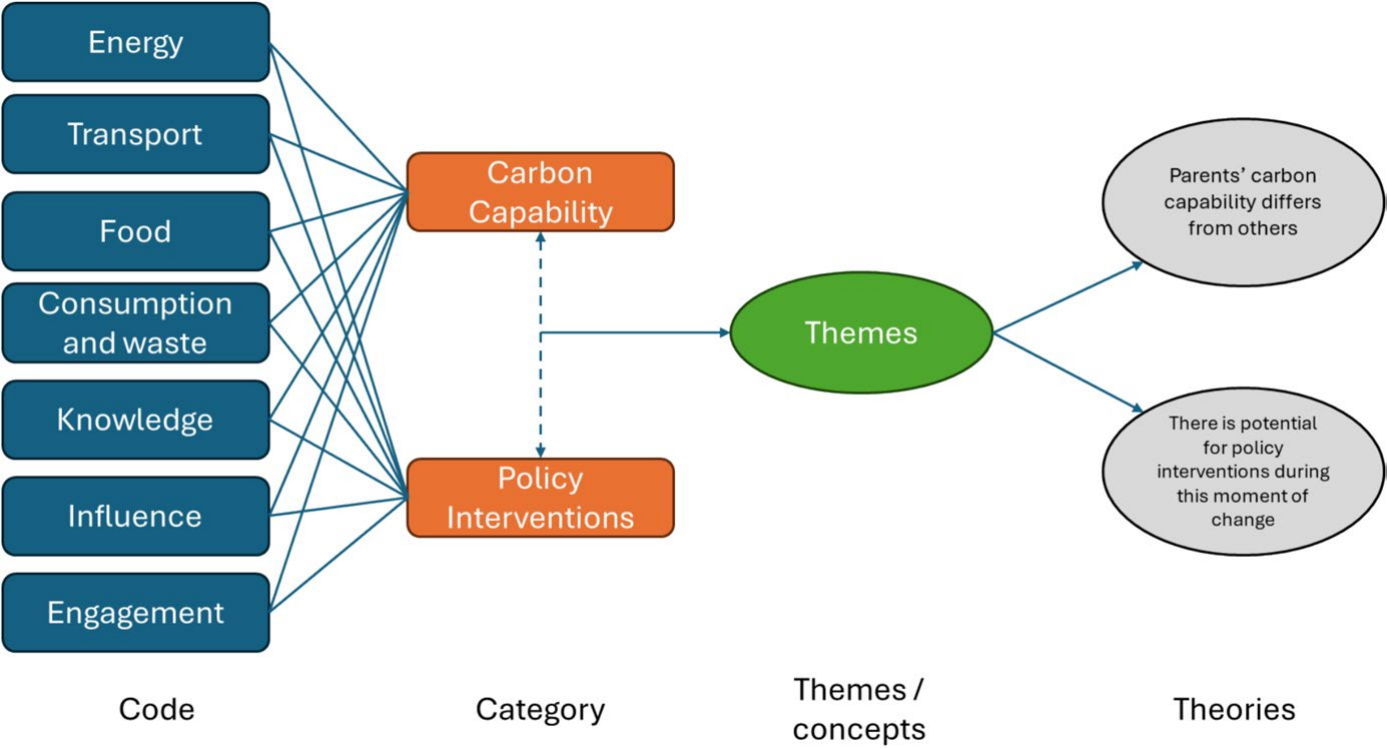
Process of coding transcribed interview data, inspired by Saldana, 2009

Qualitative data from interviews and focus groups were used primarily to address RQ2: What can be done to enhance the carbon capability of parents? Online interviews were transcribed using Microsoft Teams, while in-person interviews and focus groups were audio-recorded and transcribed by hand. Transcripts were then coded thematically using Saldana’s (2009) framework (Fig. 2). Initial codes were generated to capture key concepts within the data, which were subsequently refined through an iterative process of comparison across transcripts. Codes were then grouped into higher-order categories to develop themes relating to parental practices, barriers, and opportunities. To aid this process, colour highlighting was used to connect repeating ideas, and illustrative quotations were organised by theme in a spreadsheet. This approach enabled us to move from descriptive coding to analytical themes that informed both theoretical development and policy recommendations.

Detailed statistical outputs are provided in Appendix 2, and supplementary tables give the full results.

## Results

This section is split into two parts, corresponding with each research question. Evaluating the carbon capability of parents, the “Assessing carbon capability” section follows the structure of the framework depicted in Fig. 1, focusing on (1) the consumption domains associated with carbon emissions (energy, transport, consumption, and food); (2) the role of knowledge, judgement, and engagement with climate change; and (3) influence capabilities. We present inferential and descriptive statistics comparing parent groups and adults without young children, followed by results from the interviews for each domain. The “Support for improving the carbon capability of parents” section presents results from focus group discussions.

### Assessing carbon capability

#### Consumption domains

##### Energy in the home

Householders tend not to know, or underestimate, their energy use (Schley & DeKay, 2015). Therefore, rather than requesting consumption estimates in kWh, our survey included several questions on energy behaviours and attitudes. This included their typical home heating temperature in winter and their household’s self-reported effort to reduce gas or electricity consumption (see Appendix 1, wave 2 Q5, and Q2_2, respectively). Descriptive statistics for key consumption behaviours are detailed in Table 1.

**Table 1:** Descriptive statistics for key behaviours across the four carbon capability consumption domains. See Appendix 1 for full details of items and scales and Appendix 2 for ANOVA test results

		Group 1: parents of 0–7 only		Group 2: parents of 8–15 only		Group 3: parents of both 0–7 and 8–15		Groups 1–3: parent of under 15		Group 4: adults without young children	
		Mean	SD	Mean	SD	Mean	SD	Mean	SD	Mean	SD
Energy	Household effort made to limit of reduce gas and electricity usage	3.2	0.6	3.2	0.8	3.1	0.7	3.2	0.7	3.3	0.7
	Adjusts heating for children***	0.6	0.5	0.3	0.5	0.5	0.5	0.4	0.5	0.1	0.2
	Thermostat set point	4.4	1.5	4.3	1.3	4.1	1.2	4.3	1.4	4.2	1.3
	Frequency of conversations about saving energy***	3.2	1.7	3.3	1.6	3.5	1.4	3.3	1.6	2.8	1.6
Transport	Car usage (hours per week)***	2.5	1.0	2.7	1.0	3.0	1.0	2.7	1.0	2.2	1.0
	Frequency of conversations about reducing car usage ***	2.0	1.6	1.8	1.4	2.3	1.5	2.0	1.5	1.7	1.2
	Time spent flying for leisure	1.4	1.1	1.7	1.3	1.5	1.0	1.6	1.2	1.6	1.3
Shopping	Frequency of buying disposable items***	2.9	1.5	2.8	1.6	3.8	1.6	3.0	1.6	2.4	1.4
	Frequency of buying second-hand***	3.0	1.6	2.5	1.6	3.3	1.7	2.8	1.6	2.4	1.4
	Frequency of borrowing/renting items***	2.0	1.7	1.8	1.4	2.4	1.8	2.0	1.6	1.4	0.9
Food	Agrees that they would change their diet if they could***	3.9	1.7	3.8	1.6	3.9	1.6	3.9	1.6	3.3	1.5
	Frequency of eating red meat	4.5	1.7	4.3	1.8	4.4	1.6	4.4	1.8	4.6	1.8

^*^p < 0.05; **p < 0.01; ***p < 0.001

A comparison of groups revealed no statistically significant differences in overall household effort to limit gas and electricity use (*p* = 0.455) or thermostat set point (*p* = 0.579) (see Table S3.1 and Appendix 2 for full regression results). However, parents of young children (group 1) were significantly more likely to adjust thermostats for the sake of their children than parents of older children, with a mean score of 0.6 in group 1 compared to 0.3 in group 2 (*p* < 0.001). Regression analyses, which control for other socio-demographic factors, confirmed that parents in groups 1–3 were each more likely to report this behaviour than those without young children (see Table S3.2). When asked about recent conversations about saving energy at home, significant differences were found between groups: parents of children under 15 reported having these conversations more frequently (mean score 3.3) than adults without young children (mean score 2.8; see Table S2.1). These findings were consistent across all three analytic approaches: ANOVA was used to test for overall group differences, post-hoc tests identified which groups differed from each other, and regression models tested whether patterns remained after adjusting for confounders.

In interviews, 20 of 30 respondents said they had used more heating since having children, especially during the newborn stage, which many interviewees attributed to a perceived health-related pressure to maintain the correct temperature for babies. One focus group participant reported that “there’s this pressure to maintain the optimal temperature” (FG-6–4), while another interviewee explained that they “felt almost obliged to keep the house at just the right temperature for them” (MJ-3–3).

##### Transport behaviours

Participants reported their typical weekly car travel time (including commuting, as driver or passenger) using pre-defined categories (Appendix 1, wave 1 Q28). Although initial analysis showed a significant difference between groups, regression analysis revealed that when controlling for other variables, only group 3 (parents of mixed-age children) spent significantly more time using cars (Table S3.4).

Interviews corroborated this finding. Twenty-one of 30 interviewees reported more frequent car use after having children, citing convenience and accessibility. Driving was said to be “just so much easier” (FK-3–3) than travelling on public transport with children. Statements such as “you have to take a huge detour to use [the lift]” (ML-2–4) and “it’s just so difficult to take a buggy on the train” (FB-1) illustrate that accessibility presents a barrier to public transport for many. Despite high car dependency, we also found that some parents (groups 1 and 3) were likely to have had conversations about *reducing* car usage in the last month than adults without young children (Table S3.5).

Survey analysis showed no significant group differences in self-reported flight time for leisure travel in the past year (see wave 1 Q30 in Appendix 1 and Table S2.1 for ANOVA results), but several interviewees said that flying with young children is difficult and stressful:

It’s absolute chaos flying with two young children (FC-2–5).

It’s more stress than it’s worth (FN-1–3-5).

Some mothers reported a “*sense of mortality*” *(FD-6)* associated with the risk of flying and said safety concerns were more significant than environmental impacts. Although several interviewees expressed guilt relating to flying, only two said they typically offset their flight-related emissions. One father said flying was “expensive enough” (MH-6–3).

##### Consumption practices 

Respondents were asked how frequently they purchase disposable or single-use items on a 7-point scale (Appendix 1, wave 2 Q20_8). Results showed that adults without young children do so significantly less frequently (S2.2). This is in line with expectations, as raising children is often associated with single-use items such as nappies and wet wipes. Surprisingly however, regression analysis confounded this assumption, as group 1 was not significantly more likely to adopt these consumption behaviours when controlling for other variables, whereas groups 2 and 3 *was* (Table S3.6).

Roughly three billion nappies are thrown away annually in the UK, comprising 2–3% of all household waste (WRAP (n.d.). Several interviewees experienced guilt relating to the environmental impacts of nappy waste:

I can’t even tell you how many nappies we go through, it’s awful (FO-2–2).

Yet 19 of 30 interviewees exclusively used disposable nappies due to their “*convenience*” (FI-6–3) or “*ease of use*” (MO-2–2). Five had *tried* reusables but resorted to disposables, three use reusable nappies, and three did not say. Parents explained that the decision to use disposable or reusable nappies was made early on, after which they became “stuck in [their] ways” (FA-6–3-1). Two parents used washable wipes with disposable nappies, which helped to allay their guilt: “we use more wipes than nappies, so if we can cut wipe use down, I feel a bit better” (FF-4–1). Parents also expressed concerns with the accumulation of plastic toys:

The house is covered in little bits of plastic, it makes me so sad (MM-3–5).

One parent’s plastic guilt had led to a resolution to only buy toys from second-hand shops (MN-1–3-5). Survey evidence corroborated this: parents were more likely to buy second-hand and to borrow or rent items than adults without young children (Appendix 1, wave 2 Q20:5–8; ANOVA results in Table S2.2). Regression analyses showed that parents of younger children (group 1) were significantly likely to buy second-hand items at least once a week (Table S3.7). Furthermore, being a member of any early parent group (groups 1–3) increased the likelihood of borrowing or renting items, compared with adults without young children (Table S3.8).

##### Food and diet

There were no statistically significant differences between groups in the frequency of red meat consumption (see Table S2.1). However, regression analysis—which allows adjustment for other socio-demographic variables—revealed that parents of young children (group 1) and women were significantly more likely to agree with the statement ‘I would like to change my diet if I could’ (see Table S3.9). For example, the mean agreement score was higher among parents of young children (mean = 3.9) compared to adults without young children (mean = 3.3, *p* < 0.001). This indicates that while actual dietary behaviours such as red meat consumption may not differ, there was greater appetite among these groups for dietary change, even after socio-demographic differences were taken into account.

Interviews supported these findings, as parents reported difficulty in changing their diets as their food patterns are strongly influenced by concerns for their children’s nutrition and their preferences:

I think more about nutrition than the environment (FJ-6–7).

Our meals are largely driven by what the children will eat. (FO-2–2).

I have too much on my plate to worry about what’s on my plate (FC-2–5).

These quotations illustrate how environmental concerns compete with other priorities for parents.

#### Knowledge, values, judgement, and engagement

##### Perceptions of climate change

Our survey asked respondents about the urgency of climate change and the threat it poses to their families (see Table 2; Appendix 1). On a 5-point scale (1 = strongly disagree, 5 = strongly agree), parents of children under 15 scored a mean of 3.3 on ‘considers climate change a threat’, indicating a moderate level of concern. Adults without young children scored slightly lower (3.1). For ‘considers climate change an urgent problem’, all groups averaged 2.1, suggesting overall low perceptions of urgency. There were no significant group differences in these attitudes, and regression analysis showed that parent status and other demographics did not predict perceived urgency (Table S3.10). Thus, both parents and non-parents expressed moderate concern but low urgency, providing important context for interpreting their climate-related behaviours.

**Table 2:** Descriptive statistics for knowledge, values, judgement and engagement measures of CC. See Appendix 1 for full details of items and scales and Appendix 2 for ANOVA test results

		Group 1: parents of 0–7 only		Group 2: parents of 8–15 only		Group 3: parents of both 0–7 and 8–15		Groups 1–3: parent of under 15		Group 4: adults without young children	
		Mean	SD	Mean	SD	Mean	SD	Mean	SD	Mean	SD
Perception	Considers climate change as threat to themselves and their family	3.3	1.1	3.3	1.0	3.3	1.1	3.3	1.0	3.1	1.1
	Considers climate change an urgent problem	2.1	1.0	2.1	0.9	2.2	1.0	2.1	1.0	2.1	1.1
Knowledge	Understanding of climate change	4.0	0.6	3.9	0.6	3.9	0.8	3.9	0.7	3.9	0.6
	Frequency of seeking out information about climate change*	1.5	0.8	1.5	0.8	1.8	1.1	1.6	0.8	1.4	0.7
Citizenship	Written to politicians	0.1	0.3	0.1	0.3	0.1	0.3	0.1	0.3	0.1	0.3
	Posted on social media***	0.2	0.4	0.1	0.3	0.2	0.4	0.2	0.4	0.1	0.3
	Donated to environmental charity***	0.2	0.4	0.2	0.4	0.3	0.5	0.2	0.4	0.1	0.3

^*^p < 0.05; **p < 0.01; ***p < 0.001

In interviews, participants acknowledged the importance and urgency of climate change, but saw this as secondary to threats such as the “cost-of-living crisis” (MK-1–4-6) and, coming shortly after Russia’s invasion of Ukraine, “war” (FE-6). Nonetheless, several parents said that their concern about climate change had increased since having a child, sometimes leading to anxiety:

When you have someone else to look after, your fear of all threats increases (MI-6-7).

Interviews revealed some differences between mothers’ and fathers’ perceptions of climate change. Respondents were each asked to list up to three words that come to mind when thinking of climate change (drawing on previous research eliciting spontaneous associations; Smith & Joffe, 2012), yielding a total of 67 responses (Table 3). Both mothers and fathers most frequently associated climate change with ‘heat/sun’, but mothers were substantially more likely than fathers to mention ‘future/children’ (13 out of 15 mothers versus 6 out of 15 fathers; Fisher’s exact test, *p* = 0.021), suggesting a greater tendency among mothers to link climate change to concerns for the next generation. However, elsewhere in interviews, several fathers also discussed climate change in relation to the future of their children, often expressing a desire to protect their family. Yet many also felt out of control:

I worry what the world will look like when we are no longer around to protect them (MF-4-6).

I know it’s a threat, but I don’t really know what to do about it (ML-2-4)

**Table 3:** ‘*Which words come to mind when you think about climate change?*’

Words mentioned	Fathers (*n* = 15)	Mothers (*n* = 15)
Heat/sun	10	10
Future/children*	6	13
Fear/stress/worry	6	5
Natural resources	4	2
Carbon/greenhouse gases	3	4
Iceberg	1	1
Pressure	1	1

##### Knowledge of climate change

Survey participants were asked how well they understood climate change on a scale of 1–5 (1 = ‘never heard of this before’, 5 = ‘understand it very well’). The mean score was 3.9 for parents of children under 15 and 3.9 for adults without young children (see Table 2), indicating high self-reported knowledge across groups and suggesting a possible ceiling effect. Thus, most respondents felt fairly or very knowledgeable about climate change, with few reporting little or no understanding. However, interviews revealed that, when asked about more complex issues such as carbon offsetting or net zero, some parents acknowledged gaps in their knowledge that were not captured by their initial self-assessment:

[laughing] I’ve never heard of carbon offsetting, maybe I don’t know as much as I thought I did (MG-3-3).

We asked survey respondents how often they seek out information on climate change in a typical week, with possible responses ranging from ‘not at all’ to ‘every day’ (Appendix 1, wave 1 Q45; Table S2.1). Responses were recoded to identify frequent information seekers, defined as those seeking information at least three days per week. Binary logistic regression revealed that parents of children of mixed ages (group 3: those with both younger and older children) were significantly more likely to be frequent seekers of climate change information than other groups. Specifically, group 3 parents had higher odds of seeking information frequently compared with parents of only younger children, parents of only older children, and adults without young children (Table S3.11). This suggests that parents managing children at multiple developmental stages may be particularly engaged with climate change issues, potentially reflecting increased perceived relevance or responsibility in guiding a wider age range.

##### Citizenship

**Fig. 3:**
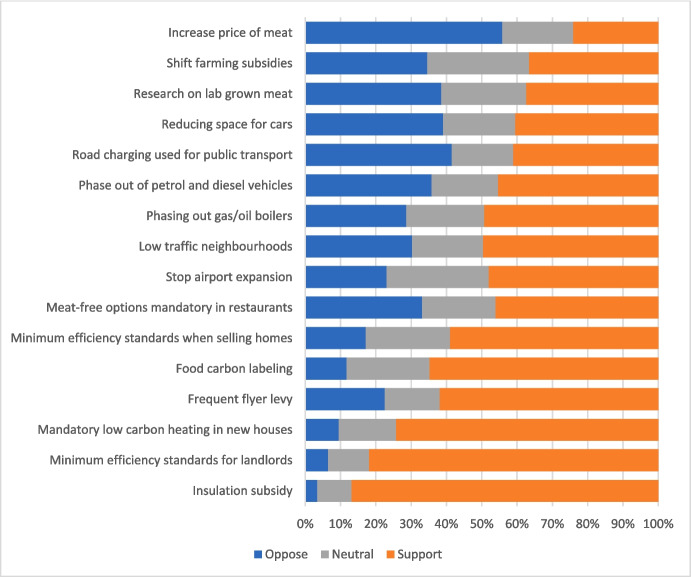
Support for climate policies among parents of young children (groups 1–3)

Survey respondents were asked the degree to which they supported 16 extant and hypothetical climate policies (Appendix 1, wave 1 Q51; Fig. 3). On a 1–7 scale, where 7 indicates high levels of support, the mean score across all 16 policies for parents (groups 1–3 combined) was 4.58. This compares with 4.39 for adults without young children, indicating that parents of children under 15 were slightly more supportive of climate policies overall (Table S2.3).

Reflecting the importance of wider engagement in the carbon capability framework (Whitmarsh et al., 2011), respondents were asked about a range of citizenship behaviours, including whether they had written to politicians or posted on social media about climate change, or donated to an environmental charity. Across groups, mean scores for these actions were all low (all groups scored near or below 0.3 on a 0–1 scale; see Table 2), indicating that direct civic engagement was uncommon among both parents and non-parents. While ANOVA tests indicated group differences for social media and donation behaviours, regression analyses showed that gender, age, and education were stronger predictors of social media activity than parenthood (Table S3.12). However, parents of older children and those with children in both age groups (groups 2 and 3) were significantly more likely to donate to environmental charities than other adults (Table S3.13), though the overall rates were still low.

**Fig. 4:**
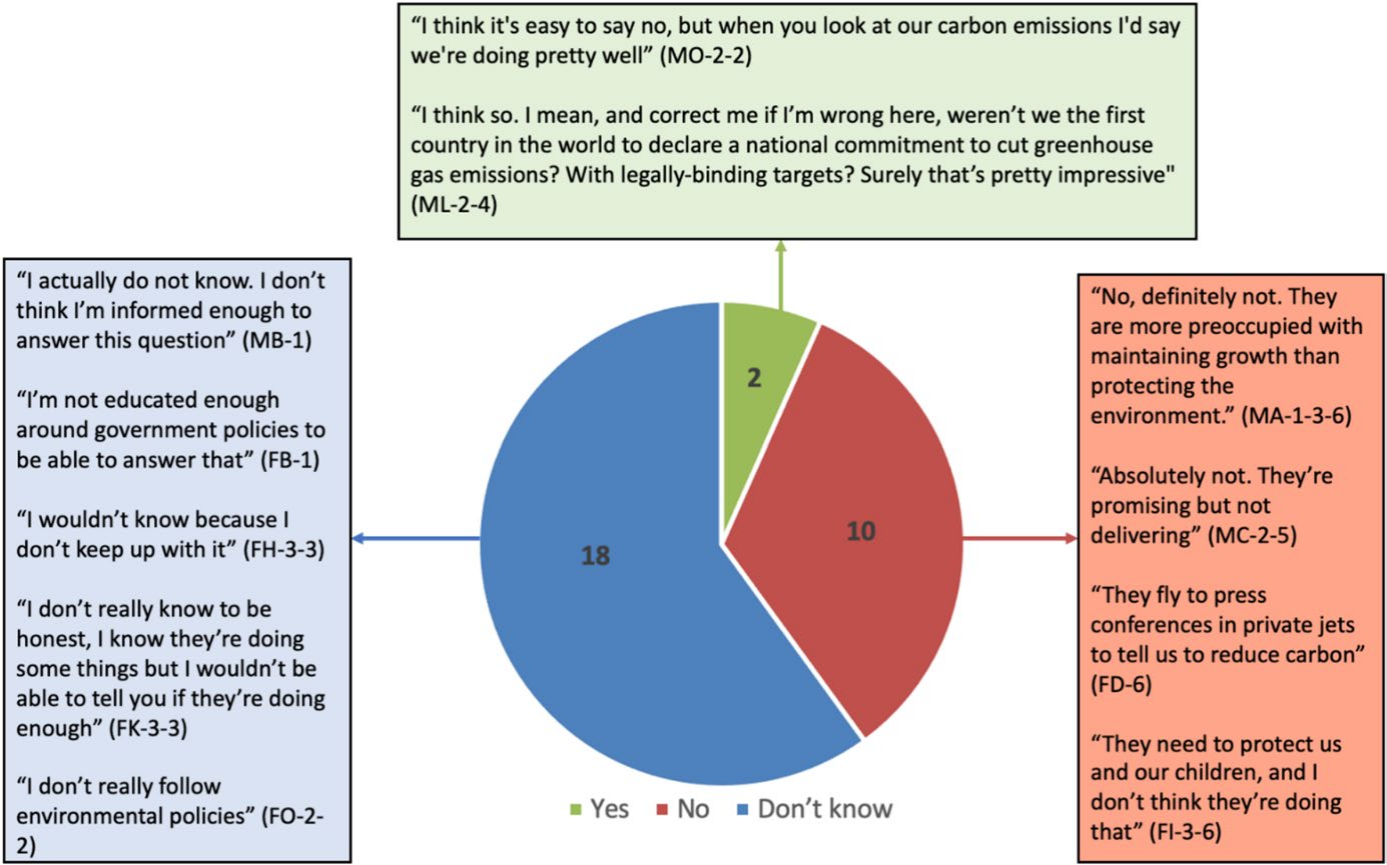
‘*Do you think the UK government is doing enough to tackle climate change?*’: selected interview quotations

Interview data suggests that low levels of engagement could be connected to a lack of knowledge, where many parents felt inadequately informed about these interventions to have strong opinions. Notably, 18 of 30 interviewees said that they were not knowledgeable enough to answer the question ‘is the UK government is doing enough to tackle climate change’ (Fig. 4). These findings demonstrate (1) that our parent interviewees were aware and willing to be honest about their limited knowledge; and (2) the value of mixed methods for revealing nuance behind survey responses.

##### Having fewer children

Interviewees were asked whether they had discussed the environmental consequences of having more children with their partners. This topic generated polarised results. Some participants strongly defended their right to have children:

I feel it’s my duty to have children (FB-1).

Other participants said they had grappled with this issue and could see it from different perspectives:

It fights with the natural instincts of humans to procreate… but we’re so far evolved that we do actually have a choice? (FG-4-6).

Five participants described coming to a ‘compromise’ of having no more than two children, citing the reproductive replacement rate:

I’ve replaced myself and that’s enough (MG-3–3).

#### Influence capability

Influence capabilities encompass both an individual’s ability to influence others, as well as their openness to influence by others. Respondents rated their own influence on their family’s climate impact from 1 (‘no influence at all’) to 5 (‘very influential’). As shown in Table 4, the mean score for parents of children under 15 was 3.0, only slightly higher than for adults without young children (2.8), indicating that most parents saw themselves as moderately influential. While the difference between group 3 (parents of both age groups) and adults without young children was statistically significant, the overall level of self-perceived influence was not high in any group.

**Table 4:** Descriptive statistics for knowledge, values, judgement and engagement measures of CC. See Appendix 1 for full details of items and scales and Appendix 2 for ANOVA test results

		Group 1: parents of 0–7 only		Group 2: parents of 8–15 only		Group 3: parents of both 0–7 and 8–15		Groups 1–3: parent of under 15		Group 4: adults without young children	
		Mean	SD	Mean	SD	Mean	SD	Mean	SD	Mean	SD
Influence	Ability to influence family	3.0	1.0	3.0	1.2	3.3	1.3	3.0	1.1	2.8	1.2
	Has tried to persuade friends	0.3	0.4	0.2	0.4	0.3	0.5	0.3	0.4	0.2	0.4
	Ease of talking: immediate family	1.7	0.9	1.7	1.1	1.7	1.0	1.7	1.0	1.8	1.0
	Ease of talking: extended family	2.1	1.0	2.2	1.0	2.1	1.1	2.1	1.0	2.1	1.0
	Ease of talking: friends	1.9	0.9	2.1	0.9	2.1	1.1	2.0	1.0	2.0	0.9
	Ease of talking: colleagues	2.1	0.9	2.2	1.0	2.1	1.1	2.2	1.0	2.1	0.9
	Ease of talking: neighbours	2.5	1.1	2.3	1.0	2.2	1.1	2.4	1.1	2.4	1.0
	Ease of talking: community members	2.3	1.0	2.5	1.0	2.3	0.9	2.4	1.0	2.4	1.0

^*^p < 0.05; **p < 0.01; ***p < 0.001

Participants were also asked if they had tried to persuade their friends to reduce their carbon footprint. Only 22% of UK citizens said they had done this or were in the process of doing this, and parent groups were no more likely than other adults to report this.

Participants rated the ease of discussing environmental issues with various groups on a 5-point scale (1 = ‘very easy, we readily talk about it’; 5 = ‘very difficult, it is awkward’). As shown in Table 4, mean scores for parents of under-15 s ranged from 1.7 (for immediate family) to 2.5 (for neighbours), indicating that these conversations were generally rated between ‘easy’ and ‘neutral’—but not ‘very easy’—even within families. Adults without young children gave similar ratings. Thus, parent groups did not find it significantly easier than other adults to discuss environmental issues, even with their immediate family. Most respondents experienced some degree of awkwardness or hesitation rather than completely open dialogue. Supporting this finding, several interviewees reported difficulty in talking with their children about environmental issues, giving two main reasons: insufficient climate knowledge and the risk of making their children “absolutely petrified” (FK-3–3) and “unable to sleep at night” (FI-3–6). However, interviewees also demonstrated acute awareness of their climate influence, which was often non-verbal:

They’re like a blank canvas… if they see you litter, they will litter. (MG-3–3).

They copy what we do. (ML-2–4).

Similarly, with respect to food and energy consumption behaviours:

They eat what we eat (FJ-6–7).

The eldest always does it [turning off lights] out of habit of seeing us do it (FL-1-4-6)

Parents also emphasised the two-way nature of climate influence and the potential for inter-generational learning. One mother said:

I’ve told her so many times to have quick showers, now she knocks on the door when I’m showering and tells me to hurry up (FD-6).

For some, their children’s environmental interests had encouraged them to learn more themselves:

My eldest knows more than me…so when a leaflet comes home in his school bag, I’ll go off and read anything I can to try and support his learning (FA-1-3-6).

Other anecdotes described children taking on responsibility to encourage their siblings and parents to conserve resources:

I always used to tell the eldest to turn the tap off when she brushes her teeth, not to waste water. Now she turns the tap off when she sees me washing my hands! (FG-4-6).

The eldest now tells his sister to turn off the lights after us telling him so many times (FA-1-3-6)

### Support for improving the carbon capability of parents

A consistent and intriguing finding from interviews was respondents’ tendency to propose their own interventions for improving the carbon capability of parents. This prompted further exploration in focus groups, where parent-led discussions primarily centred around the provision of information and awareness raising, but also touched on more structural or regulatory changes.

Participants exhibited a clear preference for educational interventions that were practical, actionable, and timed to coincide with specific ‘moments of change’ (MoCs). For instance, while information about energy, food, and sustainable transport was generally seen as more useful after the birth of a child—when parents have had time to adjust and are seeking solutions to emerging problems—nappies were considered an exception: “just one pre-natal class would be enough to make me consider changing [to reusables]” (FR-1–4). This demonstrates the critical importance of timing, as habits around nappies are established immediately after childbirth.

Beyond nappies, participants voiced a strong desire for trusted and accessible sources of guidance across other sustainability domains. One mother noted, “I would say [I know how to heat my house efficiently], but it took f***ing ages to figure it out” (FR-1–4), highlighting knowledge gaps around domestic energy use. Many echoed the need for centralised sources of practical information, suggesting a dedicated website or app for parents: “Having an app where you can get bite-sized information, recipes, tips for travelling with kids, what to do if you want to find reusables—everything in one place—would make my life much easier” (FP-1–3). The male focus group even went so far as to sketch out a mock-up interface during discussion.

Information provision through digital means was seen as most viable, given parents’ time constraints. Most group members agreed, “during breastfeeding is the only free time you get in the day” (FP-1–3), and thus valued formats that could be easily accessed on a phone. Podcasts and social media were suggested to extend the reach of any information delivered. However, participants repeatedly emphasised the importance of tailoring the channel and messenger to the message: “Antenatal classes are brilliant for showing you nappies, but if you want parents to believe you about science, you need that to come from the school or nursery” (FQ-3–5). Government was seen as the most trusted authority for energy-saving advice, while nurseries and schools were preferred conduits for climate science.

Discussion also extended to more structural interventions. The idea of a new tax on aviation was met with a diversity of views, with some seeing it as necessary for equality—“The only fair way would be an income-based tax, otherwise it just punishes those who want to see family abroad” (MP-6)—while others worried it would disproportionately affect lower-income families. Many participants suggested that instead of simply penalising behaviour, government campaigns should “advertise the beauty of the UK, make it aspirational to holiday here rather than abroad” (FR-1–4). There was also appetite for policies targeting high-carbon consumption. “I would happily give up meat if eating less would make a big difference”, one participant reflected (MQ-1–3), while others supported making plastic toys more expensive to incentivise charity shop purchases: “It’s crazy the amount of plastic we’re surrounded by. If there was a way to make toys last longer, or to make it cost more to buy new, more people would shop second-hand” (FQ-3–5).

Furthermore, participants recognised the sensitive potential for guidance on family planning (the ‘carbon legacy’ of children): “If parents knew the impact [of having another child], some would think twice, but obviously it’s a personal decision.” While divisive, this underscores the openness among some to broader conversations around the environmental consequences of family size, provided such discussions are handled carefully and non-judgementally.

Taken together, these findings paint a nuanced picture of parents’ priorities and perceived barriers. Focus group discussions reveal that while parents are receptive to interventions across multiple domains, the success of such measures depends on both sensible timing and the perceived legitimacy of the message and its messenger. Policy initiatives should therefore be multifaceted, combining targeted information at key life transitions with more systemic and structural changes to support sustainable habits over the long term.

## Discussion

Our findings indicated that carbon capability among parents of children under 15 in the UK was limited, challenging the idea of the ‘green parenthood effect’ (Shrum et al., 2023). This study contributes to the debate on the potential of parenthood to drive sustainability by demonstrating that (a) parents’ consumption habits were often no better, and in some cases worse, than those without children; but (b) parents are uniquely positioned to act as catalysts for intergenerational, pro-environmental change.

In many ways, the environmental impacts of parents mirror those of the wider UK population, which has been shown to have limited carbon capability (Hampton & Whitmarsh, 2024). This parents in this study made no more effort to save energy in the home and were more likely to increase temperature to accommodate their children’s needs. They relied on cars for transport, paid little attention to their dietary impacts, and produced significant quantities of waste. They reported low levels of knowledge and were aware that this limits their ability to support children’s learning. Time constraints and the prioritisation of convenience further exacerbated these patterns. Whereas previous research observed that parents of young children often reduced travel due to a more geographically confined lifestyle (Thompson et al., 2011), our findings indicated that the inaccessibility of public transport and convenience of cars meant that parents spent more time using private vehicles than adults without young children. Another barrier was competing priorities, including children’s health and development (Burningham & Venn, 2020). Interviews and focus groups revealed the emotionally charged nature of climate change and parenting, supporting Gaziulusoy’s (2020) findings that having a child can lead to heightened climate anxiety.

However, neither awareness of their own environmental impacts nor fear for their children’s future appeared to be significant motivators for pro-environmental behaviour change. Our study corroborated evidence that guilt is a poor motivator (Lickel et al., 2014; Trojanowski et al., 2020). Despite widespread guilt among parents, respondents tended to justify their behaviours rather than express willingness to change. They were aware that their lifestyles and parenting decisions were not commensurate with climate action and cited various personal and social barriers. They also emphasised the lack of government action to make low-carbon choices more widespread.

Despite these challenges, our sample of parents also possessed a unique capacity to *influence* and *be influenced* on environmental issues. Although self-reporting low levels of knowledge, survey data suggested that parents of children under 15 actively sought out information about climate change. Parent–child dynamics have been shown to sometimes promote environmentally conscious values within the family (Lawson et al., 2019). However, interviewees reported that a barrier to having more climate conversations was the fear of scaring their children. Eager to learn and support their children, parents would welcome help with having these conversations (Gaziulusoy, 2020; Nche et al., 2019). Initiatives such as *Parents for the Future* (( n.d.)) address this need but are limited in extent. More structured support through schools or antenatal classes is needed.

Parents participating in focus groups emphasised the importance of timing information provision with key stages in family development. Examples included school newsletters or flyers in school bags, family homework, and developing a smart-phone application (O’Brien, 2007). Embedding climate change in the school curriculum can help raise knowledge and leverage two-way influence capabilities (Pearce et al., 2020). However, many behavioural changes are unresponsive to information provision (Vlasceanu et al., 2024), and our findings have shown that awareness is insufficient to drive behavioural change alone. Other interventions emerged from our findings, too. Parents who highlighted the inaccessibility of public transport identified the need for more ‘buggy-friendly’ train and tube stations. Some topics generated contentious debate: participants were supportive of a tax on the most impactful foods to motivate more environmentally friendly diet choices, and others said that a carbon tax on aviation would make them think carefully about taking their family on foreign holidays. The topic of having fewer children polarised opinion. Our findings suggest that supporting parents to become more carbon capable requires multiple forms of intervention, implemented over many years.

## Conclusion

Parents represent a critical demographic in the effort to enhance societal carbon capability. This study addresses the nuanced role of early parenthood (parents of children under 15), and found that while parental lifestyles in our sample were not always aligned with climate action, parents are uniquely positioned to cultivate environmental values and enhance carbon capability in the next generation. Building on previous literature, we developed a theoretical model of carbon capability for parents with three main elements: (1) family practices including energy, transport, food, and consumption; (2) knowledge, engagement, and moral judgement; and (3) influence. Our findings suggested that parents of children under 15 in the UK performed poorly against the first two elements. Parental responsibilities often led to behaviours which—on the whole—increased parents’ carbon footprints. We found relatively high levels of awareness among parents of the environmental consequences of these actions, accompanied by widespread guilt. However, these were not motivators for change.

Despite significant potential for positive climate influence on others, including their children, parents in our study rarely realised it. Low self-perceived knowledge and a fear of scaring their children often made them reluctant to talk about climate change, although notable exceptions demonstrate the power of two-way influence between parent and child. Our study supports existing evidence that children’s exposure to climate education encourages parents to develop their own knowledge and awareness (Lawson et al., 2018). Parents in our sample were open to learning and preferred information provision as a means of intervention. However, information provision alone is insufficient to boost parents’ carbon capability and must be coupled with policy measures which make low carbon choices more appealing, affordable, and convenient. If provided with a mix of guidance, incentives, and structural reforms, parents can help to shape the future generation, moulding them into resilient, knowledgeable, and proactive climate citizens. There is a need for governments at all scales to focus on improving the capability of parents of children under 15. The potential is not only to cut emissions from some of the highest consumers in society, but to influence the habits, attitudes, and behaviours of the next generation.

This research advances the study of carbon capability both theoretically and empirically, applying this concept to parents for the first time. It moves beyond the methodological limitations of previous studies by incorporating qualitative methods (in-depth interviews and focus groups) alongside a large survey. However, it is important to acknowledge the limitations of our sample. Although the survey was geographically representative across regions of the UK, attrition between survey waves resulted in a final sample that was more likely to be older, female, and more highly educated. Rim weighting was applied to mitigate these effects and better reflect the national population profile; however, some perspectives—particularly those of younger, less educated, or male respondents—may remain underrepresented, and caution is warranted in generalising the findings. Moreover, our qualitative data is based on a limited number of in-depth interviews (*n* = 30) and two focus groups with a small number of participants (*n* = 7), which limits the generalisability of these findings. In addition, interviewees were largely recruited from London and the south-east, where average incomes are higher. These demographic skews may mean our findings slightly overemphasise the attitudes and barriers experienced by older, more highly educated or female parents, who are often both more engaged with climate issues and potentially more self-reflective in qualitative research. As a result, the challenges and opportunities for increasing carbon capability among younger, male, or less-educated parents—who may have different constraints, priorities, and communication preferences—could be underrepresented, highlighting the need for future work to more fully capture this diversity.

While this study was conducted in the UK, many of the challenges and opportunities identified are likely to be relevant in other contexts. The difficulties of balancing parental responsibilities with environmental concerns, the role of time constraints, the desire for convenience, and the potential for parent–child influence are all factors that are likely to shape parental behaviours across different cultures and societies. These will vary depending on cultural norms, economic conditions, and policy environments, and there is potential for future research to explore the carbon capability of parents in different contexts. Further research is needed in the Global South, and to compare countries.

There remains a significant lack of research and evidence relating to parenting and climate change. Perhaps more concerning is the lack of policy to enhance parents’ carbon capabilities. Parents should not be taught about climate breakdown by their young children. Adults have far greater responsibility—as voters, consumers, and primary carbon emitters—and should be equipped to guide and support the next generation, not depend on them for critical knowledge. It is incumbent on the academic and policymaking communities to support parents to become more carbon capable and to raise a new generation of climate citizens.

## Supplementary information

Below is the link to the electronic supplementary material.

ESM1 (XLSX 90.9 KB)

ESM2 (DOCX 25.1 KB)

ESM3 (DOCX 48.3 KB)

ESM4 (DOCX 20.6 KB)

ESM5 (DOCX 18.8 KB)

## Data Availability

Data has been uploaded to the University of Bath’s research repository, under embargo until the conclusion of the wider research project. Files will remain under embargo until 22 December 2025. The DOI is 10.15125/BATH-01362.

## Footnotes

1 Survey data were analysed using SPSS (version 28.0). Homogeneity of variances was checked with Levene’s test; standard ANOVA was applied when the assumption held, with Welch’s ANOVA used otherwise. A post hoc power analysis conducted in G*Power (version 3.1) showed that, with *α* = 0.05, numerator df = 3, and denominator df = 997, our ANOVA had near-perfect power (0.999999) to detect medium effects (*f* = 0.25). Regression analyses followed the guideline of at least 10 cases per predictor variable (Tabachnick and Fidell 2012) to ensure adequate power.
