# Supplementary material for: Parenting and climate change: assessing carbon capability in early parenthood
Source: Popul Environ. 2025 Sep 25;47(4):34. doi: 10.1007/s11111-025-00506-6 (PMC12464131; doi:10.1007/s11111-025-00506-6)
Supplement: Supplementary file 2 — (DOCX 25.1 KB) [file 11111_2025_506_MOESM2_ESM.docx]

# Supplementary Material: Appendix S2, anova and t-test

## Table S2.1: ANOVA test results

| **Survey question** | **Sum of squares** | **df** | **Mean square** | **F-value** | **Sig.** | **Eta squared ($\eta^2$)** |
| --- | --- | --- | --- | --- | --- | --- |
| Consumption behaviours | | | | | |  |
| Household effort made to limit of reduce gas and electricity usage | 1.367 | 3 | .456 | .873 | .455 | .003 |
| Thermostat set point | 3.005 | 3 | 1.002 | .579 | .579 | .002 |
| Frequency of conversations about saving energy *** | 49.215 | 3 | 16.405 | 6.667 | <.001 | .020 |
| Car usage (hours per week *** | 39.854 | 3 | 13.285 | 13.792 | <.001 | .040 |
| Time spent flying for leisure | 4.914 | 3 | 1.638 | .970 | .406 | .003 |
| Agrees that they would change their diet if they could*** | 60.461 | 3 | 20.154 | 8.247 | <.001 | .024 |
| Frequency of eating red meat | 5.690 | 3 | 1.897 | .605 | .612 | .002 |
| Knowledge, values, judgement and engagement | | | | | |  |
| Considers climate change as threat to themselves and their family | 8.735 | 3 | 2.912 | 2.409 | .066 | .007 |
| Considers climate change an urgent problem | .287 | 3 | .096 | .082 | .970 | .000 |
| Understanding of climate change | .136 | 3 | .045 | .110 | .955 | .000 |
| Frequency of seeking out information about climate change* | 536.764 | 3 | 178.921 | 3.050 | .028 | .009 |
| Written to politicians | .140 | 3 | .047 | .627 | .597 | .002 |
| Influence | | | | | |  |
| Has tried to persuade friends to reduce emissions | .587 | 3 | .196 | 1.145 | .330 | .003 |
| Ease of talking about environmental issues with immediate family** | 2.091 | 3 | 0.697 | 0.744 | 0.526 | .002 |
| Ease of talking about environmental issues with extended family | 0.769 | 3 | 0.256 | 0.257 | 0.857 | .001 |
| Ease of talking about environmental issues with friends* | 2.165 | 3 | 0.722 | 0.835 | 0.475 | .003 |
| Ease of talking about environmental issues with colleagues | 0.628 | 3 | 0.209 | 0.228 | 0.877 | .001 |
| Ease of talking about environmental issues with neighbours | 1.337 | 3 | 0.446 | 0.438 | 0.726 | .002 |
| Ease of talking about environmental issues with members of communities they’re involved in* | 1.829 | 3 | 0.61 | 0.625 | 0.599 | .003 |

*p<0.05; **p<0.01; ***p<0.001

## Table S2.2 - Robust Tests of Equality of Means (Welch test)

| **Variable** | **Statistic^a^** | **df1** | **df2** | **Sig.** |
| --- | --- | --- | --- | --- |
| Adjusts heating for children*** | 43.81 | 3 | 102.718 | <.001 |
| Frequency of talk about reducing car use** | 4.341 | 3 | 108.198 | 0.006 |
| Frequency of buying disposable items*** | 11.155 | 3 | 110.393 | <.001 |
| Frequency of buying second-hand*** | 7.209 | 3 | 109.731 | <.001 |
| Frequency of borrowing / renting items*** | 9.519 | 3 | 104.342 | <.001 |
| Posted on social media | 2.452 | 3 | 107.693 | 0.067 |
| Donated to charity** | 5.146 | 3 | 107.054 | 0.002 |
| Ability to influence family to reduce emissions* | 3.061 | 3 | 112.991 | 0.031 |
| *p<0.05; **p<0.01; ***p<0.001  a. Asymptotically F distributed. |  |  |  |  |

## Table S2.3

Average support for 16 climate policies, based on 7 point Likert scale (1 = strongly oppose, 7 = strongly support)

| **Group** | **Mean** | **Std. Deviation** | **N** | **t-value** | **df** | **p-value** |
| --- | --- | --- | --- | --- | --- | --- |
| Parents | 4.58 | 1.11 | 228 | -2.19 | 397 | 0.029 |
| Adults without young children | 4.39 | 1.20 | 773 |  |  |  |
