# Supplementary material for: Parenting and climate change: assessing carbon capability in early parenthood
Source: Popul Environ. 2025 Sep 25;47(4):34. doi: 10.1007/s11111-025-00506-6 (PMC12464131; doi:10.1007/s11111-025-00506-6)
Supplement: Supplementary file 3 — (DOCX 48.3 KB) [file 11111_2025_506_MOESM3_ESM.docx]

# Supplementary Material: Appendix S3. Regression tables.

All tables in this appendix display the results of binary logistic regressions.

#### Table S3.1: ‘How much effort do you make to reduce gas and electricity use?’: some / a lot / a great deal (Q5_w2)

| **Variable** | **B** | **S.E.** | | **df** | **Sig.** | | **Exp(B)** | **95% C.I. for Exp(B)** | |
| --- | --- | --- | --- | --- | --- | --- | --- | --- | --- |
|  |  | |  |  | |  |  | *Lower* | *Upper* |
| Gender | -.047 | | .419 | 1 | | .911 | .954 | .420 | 2.171 |
| Age | .121 | | .167 | 1 | | .469 | 1.129 | .813 | 1.568 |
| Education | -.103 | | .351 | 1 | | .770 | .902 | .454 | 1.795 |
| Parent of 0-7 | .871 | | 1.060 | 1 | | .411 | 2.390 | .299 | 19.101 |
| Parent of 8-15 | -.527 | | .532 | 1 | | .322 | .590 | .208 | 1.675 |
| Parent of both 0-7 and 8-15 | .043 | | 1.064 | 1 | | .968 | 1.044 | .130 | 8.395 |

#### Table S3.2: ‘Do you adjust your heating because of children, infants or babies?’: yes (Q2_2_w2)

| **Variable** | **B** | **S.E.** | | **df** | **Sig.** | | **Exp(B)** | **95% C.I. for Exp(B)** | |
| --- | --- | --- | --- | --- | --- | --- | --- | --- | --- |
|  |  | |  |  | |  |  | *Lower* | *Upper* |
| Gender | .199 | | .224 | 1 | | .375 | 1.220 | .786 | 1.894 |
| Age | -1.712 | | .894 | 1 | | .056 | .181 | .031 | 1.041 |
| Education | 2.719 | | 1.624 | 1 | | .094 | 15.166 | .629 | 365.724 |
| Parent of 0-7 | 2.992 | | .304 | 1 | | <.001 | 19.932 | 10.975 | 36.198 |
| Parent of 8-15 | 2.033 | | .301 | 1 | | <.001 | 7.639 | 4.234 | 13.782 |
| Parent of both 0-7 and 8-15 | 2.894 | | .407 | 1 | | <.001 | 18.064 | 8.142 | 40.079 |

#### Table S3.3: Climate conversations: ‘Have you spoken about saving energy in the home in the last month?’: yes (Q24_6_w2)

| **Variable** | **B** | **S.E.** | | **df** | **Sig.** | | **Exp(B)** | **95% C.I. for Exp(B)** | |
| --- | --- | --- | --- | --- | --- | --- | --- | --- | --- |
|  |  | |  |  | |  |  | *Lower* | *Upper* |
| Gender | .209 | | .142 | 1 | | .139 | 1.233 | .934 | 1.627 |
| Age | -.350 | | .582 | 1 | | .547 | .704 | .225 | 2.203 |
| Education | .862 | | .927 | 1 | | .352 | 2.369 | .385 | 14.582 |
| Parent of 0-7 | .595 | | .254 | 1 | | .019 | 1.813 | 1.102 | 2.983 |
| Parent of 8-15 | .539 | | .222 | 1 | | .015 | 1.714 | 1.109 | 2.651 |
| Parent of both 0-7 and 8-15 | .907 | | .356 | 1 | | .011 | 2.477 | 1.232 | 4.979 |

#### Table S3.4: ‘At the moment, how many hours a week do you spend in a car for personal use, including commuting (either as driver or passenger)?’: more than 5 (Q28_w1)

| **Variable** | **B** | **S.E.** | | **df** | **Sig.** | | **Exp(B)** | **95% C.I. for Exp(B)** | |
| --- | --- | --- | --- | --- | --- | --- | --- | --- | --- |
|  |  | |  |  | |  |  | *Lower* | *Upper* |
| Gender | -.394 | | .196 | 1 | | .045 | .674 | .459 | .991 |
| Age | 1.505 | | .869 | 1 | | .083 | 4.505 | .820 | 24.759 |
| Education | -1.559 | | 1.321 | 1 | | .238 | .210 | .016 | 2.804 |
| Parent of 0-7 | .440 | | .330 | 1 | | .183 | 1.552 | .813 | 2.965 |
| Parent of 8-15 | .057 | | .304 | 1 | | .851 | 1.059 | .584 | 1.920 |
| Parent of both 0-7 and 8-15 | .904 | | .400 | 1 | | .024 | 2.470 | 1.129 | 5.406 |

#### Table S3.5: Climate conversations: ‘Have you spoken about reducing car use in the last month?: yes (Q24_5_w2)

| **Variable** | **B** | **S.E.** | | **df** | **Sig.** | | **Exp(B)** | **95% C.I. for Exp(B)** | |
| --- | --- | --- | --- | --- | --- | --- | --- | --- | --- |
|  |  | |  |  | |  |  | *Lower* | *Upper* |
| Gender | .017 | | .212 | 1 | | .936 | 1.017 | .671 | 1.541 |
| Age | -1.472 | | .785 | 1 | | .061 | .230 | .049 | 1.068 |
| Education | -1.850 | | 1.429 | 1 | | .195 | .157 | .010 | 2.588 |
| Parent of 0-7 | .647 | | .329 | 1 | | .048 | .048 | 1.005 | 3.630 |
| Parent of 8-15 | .491 | | .322 | 1 | | .127 | .127 | .869 | 3.070 |
| Parent of both 0-7 and 8-15 | .898 | | .444 | 1 | | .043 | .043 | 1.028 | 5.865 |

#### Table S3.6: Buys disposable items (coffee cups, plastic bottles, cans, face masks, wet wipes) at least once a week (Q20_8_w2)

| **Variable** | **B** | **S.E.** | | **df** | **Sig.** | | **Exp(B)** | **95% C.I. for Exp(B)** | |
| --- | --- | --- | --- | --- | --- | --- | --- | --- | --- |
|  |  | |  |  | |  |  | *Lower* | *Upper* |
| Gender | -.326 | | .193 | 1 | | .092 | .722 | .494 | 1.054 |
| Age | -1.619 | | .716 | 1 | | .024 | .198 | .049 | .806 |
| Education | 1.608 | | 1.351 | 1 | | .234 | 4.992 | .353 | 70.558 |
| Parent of 0-7 | .118 | | .342 | 1 | | .730 | 1.125 | .576 | 2.199 |
| Parent of 8-15 | .582 | | .285 | 1 | | .041 | 1.790 | 1.024 | 3.129 |
| Parent of both 0-7 and 8-15 | 1.393 | | .381 | 1 | | <.001 | 4.027 | 1.908 | 8.502 |

#### Table S3.7: Buys second-hand items at least once a week (Q20_5_w2)

| **Variable** | **B** | **S.E.** | | **df** | **Sig.** | | **Exp(B)** | **95% C.I. for Exp(B)** | |
| --- | --- | --- | --- | --- | --- | --- | --- | --- | --- |
|  |  | |  |  | |  |  | *Lower* | *Upper* |
| Gender | -.027 | | .224 | 1 | | .905 | .973 | .627 | 1.510 |
| Age | .164 | | .851 | 1 | | .847 | 1.178 | .222 | 6.244 |
| Education | .044 | | 1.533 | 1 | | .977 | 1.045 | .052 | 21.109 |
| Parent of 0-7 | .738 | | .327 | 1 | | .024 | 2.091 | 1.102 | 3.968 |
| Parent of 8-15 | .133 | | .340 | 1 | | .695 | 1.143 | .586 | 2.226 |
| Parent of both 0-7 and 8-15 | .776 | | .440 | 1 | | .078 | 2.173 | .917 | 5.151 |

#### Table S3.8: Borrows or rents items (e.g. tools, toys) at least once a week (Q20_6_w2)

| **Variable** | **B** | **S.E.** | | **df** | **Sig.** | | **Exp(B)** | **95% C.I. for Exp(B)** | |
| --- | --- | --- | --- | --- | --- | --- | --- | --- | --- |
|  |  | |  |  | |  |  | *Lower* | *Upper* |
| Gender | -.156 | | .324 | 1 | | .631 | .856 | .453 | 1.616 |
| Age | -1.540 | | 1.143 | 1 | | .178 | .214 | .023 | 2.014 |
| Education | 3.468 | | 4.046 | 1 | | .391 | 32.061 | .021 | 89189.050 |
| Parent of 0-7 | 1.060 | | .424 | 1 | | .012 | 2.888 | 1.259 | 6.624 |
| Parent of 8-15 | .894 | | .447 | 1 | | .045 | 2.446 | 1.018 | 5.875 |
| Parent of both 0-7 and 8-15 | 1.803 | | .500 | 1 | | <.001 | 6.067 | 2.278 | 16.158 |

#### Table S3.9: ‘I would like to change my diet if I could’: agree. (Q14_14_w2)

| **Variable** | **B** | **S.E.** | | **df** | **Sig.** | | **Exp(B)** | **95% C.I. for Exp(B)** | |
| --- | --- | --- | --- | --- | --- | --- | --- | --- | --- |
|  |  | |  |  | |  |  | *Lower* | *Upper* |
| Gender | .361 | | .163 | 1 | | .027 | 1.435 | 1.041 | 1.977 |
| Age | .157 | | .627 | 1 | | .802 | 1.170 | .342 | 3.999 |
| Education | -1.317 | | 1.083 | 1 | | .224 | .268 | .032 | 2.239 |
| Parent of 0-7 | .521 | | .265 | 1 | | .049 | 1.684 | 1.002 | 2.830 |
| Parent of 8-15 | .321 | | .239 | 1 | | .178 | 1.379 | .864 | 2.202 |
| Parent of both 0-7 and 8-15 | .625 | | .366 | 1 | | .088 | 1.869 | .912 | 3.831 |

#### Table S3.10: Considers climate change to be an urgent problem (Q43_w1)

| **Variable** | **B** | **S.E.** | | **df** | **Sig.** | | **Exp(B)** | **95% C.I. for Exp(B)** | |
| --- | --- | --- | --- | --- | --- | --- | --- | --- | --- |
|  |  | |  |  | |  |  | *Lower* | *Upper* |
| Gender | .154 | | .218 | 1 | | .479 | 1.167 | .761 | 1.788 |
| Age | -2.155 | | 1.193 | 1 | | .071 | .116 | .011 | 1.201 |
| Education | .004 | | 1.433 | 1 | | .998 | 1.004 | .060 | 16.667 |
| Parent of 0-7 | .027 | | .468 | 1 | | .955 | 1.027 | .411 | 2.568 |
| Parent of 8-15 | .541 | | .425 | 1 | | .203 | 1.219 | .746 | 3.956 |
| Parent of both 0-7 and 8-15 | .062 | | .636 | 1 | | .923 | 1.064 | .306 | 3.699 |

#### Table S3.11: Seeks out information on issues related to climate change at least three days a week (Q44_w1)

| **Variable** | **B** | **S.E.** | | **df** | **Sig.** | | **Exp(B)** | **95% C.I. for Exp(B)** | |
| --- | --- | --- | --- | --- | --- | --- | --- | --- | --- |
|  |  | |  |  | |  |  | *Lower* | *Upper* |
| Gender | -.387 | | .256 | 1 | | .131 | .679 | .411 | 1.122 |
| Age | -.793 | | .980 | 1 | | .418 | .452 | .066 | 3.086 |
| Education | -2.853 | | 1.800 | 1 | | .113 | .058 | .002 | 1.964 |
| Parent of 0-7 | -.116 | | .471 | 1 | | .805 | .890 | .354 | 2.240 |
| Parent of 8-15 | .113 | | .407 | 1 | | .781 | 1.119 | .505 | 2.483 |
| Parent of both 0-7 and 8-15 | .996 | | .482 | 1 | | .039 | 2.708 | 1.052 | 6.972 |

#### Table S3.12: Has posted on social media about climate change (Q27_8_w2)

| **Variable** | **B** | **S.E.** | | **df** | **Sig.** | | **Exp(B)** | **95% C.I. for Exp(B)** | |
| --- | --- | --- | --- | --- | --- | --- | --- | --- | --- |
|  |  | |  |  | |  |  | *Lower* | *Upper* |
| Gender | -.423 | | .222 | 1 | | .056 | .655 | .425 | 1.012 |
| Age | -.331 | | .086 | 1 | | <.001 | .718 | .607 | .850 |
| Education | .488 | | .187 | 1 | | .009 | 1.629 | 1.129 | 2.351 |
| Parent of 0-7 | .224 | | .341 | 1 | | .512 | 1.250 | .641 | 2.439 |
| Parent of 8-15 | -.055 | | .328 | 1 | | .868 | .947 | .498 | 1.801 |
| Parent of both 0-7 and 8-15 | .491 | | .435 | 1 | | .259 | 1.633 | .696 | 3.831 |

#### Table S3.13: Has donated to an environmental charity (Q27_9_w2)

| **Variable** | **B** | **S.E.** | | **df** | **Sig.** | | **Exp(B)** | **95% C.I. for Exp(B)** | |
| --- | --- | --- | --- | --- | --- | --- | --- | --- | --- |
|  |  | |  |  | |  |  | *Lower* | *Upper* |
| Gender | -.581 | | .200 | 1 | | .004 | .560 | .378 | .827 |
| Age | -.143 | | .078 | 1 | | .065 | .867 | .745 | 1.009 |
| Education | .760 | | .166 | 1 | | <.001 | 2.139 | 1.544 | 2.962 |
| Parent of 0-7 | .446 | | .320 | 1 | | .163 | 1.561 | .835 | 2.922 |
| Parent of 8-15 | .604 | | .267 | 1 | | .024 | 1.829 | 1.084 | 3.085 |
| Parent of both 0-7 and 8-15 | .720 | | .412 | 1 | | .081 | 2.054 | .916 | 4.607 |
