# Supplementary material for: Parenting and climate change: assessing carbon capability in early parenthood
Source: Popul Environ. 2025 Sep 25;47(4):34. doi: 10.1007/s11111-025-00506-6 (PMC12464131; doi:10.1007/s11111-025-00506-6)
Supplement: Supplementary file 4 — (DOCX 20.6 KB) [file 11111_2025_506_MOESM4_ESM.docx]

# Supplementary Material: Appendix 4. Participant details

### Survey participant characteristics (attrition analysis)

| Variable | Characteristics | UK percentage | Frequency (n=1001) | Percent |
| --- | --- | --- | --- | --- |
| Sex | Male | 49.6 | 462 | 46.2 |
|  | Female | 50.3 | 539 | 53.8 |
| Age | 18-24 | 12.0 | 24 | 2.4 |
|  | 25-34 | 17.1 | 140 | 14.1 |
|  | 35-44 | 17.9 | 168 | 16.8 |
|  | 45-54 | 17.7 | 209 | 20.9 |
|  | 55-64 | 15.1 | 192 | 19.2 |
|  | 65+ | 20.2 | 268 | 26.8 |
| Education | Low (no formal qualifications) | 37.0 | 211 | 21.1 |
|  | Medium (A-level or equivalent) | 36.0 | 554 | 55.3 |
|  | High (Degree or equivalent) | 27.0 | 236 | 23.6 |
| Ethnicity | White or White British | 87.1 | 847 | 84.6 |
|  | Asian or Asian British | 7.0 | 64 | 6.4 |
|  | Black or Black British | 3.0 | 18 | 1.8 |
|  | Mixed | 2.0 | 16 | 1.6 |
|  | Other | 0.9 | 6 | 0.6 |
| Region | East of England | 9.3 | 97 | 7.2 |
|  | East Midlands | 7.3 | 72 | 9.7 |
|  | London | 12.9 | 120 | 12.0 |
|  | North East | 4.2 | 42 | 4.2 |
|  | North West | 11.3 | 107 | 10.7 |
|  | Northern Ireland | 2.6 | 31 | 3.1 |
|  | Scotland | 7.9 | 88 | 8.8 |
|  | South East | 13.7 | 146 | 14.6 |
|  | South West | 8.6 | 87 | 8.7 |
|  | Wales | 4.9 | 45 | 4.5 |
|  | West Midlands | 8.8 | 74 | 7.4 |
|  | Yorkshire & Humberside | 8.4 | 92 | 9.2 |

### Interview and focus group participants

| Participant code | Gender | Age |
| --- | --- | --- |
| FA-6-3-1 | Female | 25-44 |
| MA-6-3-1 | Male | 25-44 |
| MB-1 | Male | 25-44 |
| FB-1 | Female | 25-44 |
| FC-2-5 | Female | 25-44 |
| MC-2-5 | Males | 25-44 |
| FD-6 | Female | 45-64 |
| FE-6 | Female | 25-44 |
| MD-2 | Male | 25-44 |
| FF-4-1 | Female | 25-44 |
| ME-4-1 | Male | 25-44 |
| FG-6-4 | Female | 25-44 |
| MF-6-4 | Male | 25-44 |
| FH-3-3 | Female | 25-44 |
| MG-3-3 | Male | 25-44 |
| FI-6-3 | Female | 25-44 |
| MH-6-3 | Male | 25-44 |
| FJ-6-7 | Female | 45-64 |
| MI-6-7 | Male | 45-64 |
| MJ-3-3 | Male | 25-44 |
| FK-3-3 | Female | 25-44 |
| MK-1-4-6 | Male | 25-44 |
| FL-1-4-6 | Female | 25-44 |
| ML-2-4 | Male | 25-44 |
| FM-2-4 | Male | 25-44 |
| MM-3-5 | Male | 45-64 |
| MN-1-3-5 | Male | 25-44 |
| FN-1-3-5 | Female | 25-44 |
| FO-2-2 | Female | 25-44 |
| MO-2-2 | Male | 25-44 |
| MP-6 | Male | 45-64 |
| MQ-1-3 | Male | 25-44 |
| MR-1-4 | Male | 25-44 |
| MS-2 | Male | 25-44 |
| FP-1-3 | Female | 25-44 |
| FQ-3-5 | Female | 25-44 |
| FR-1-4 | Female | 25-44 |
