# Supplementary material for: Parenting and climate change: assessing carbon capability in early parenthood
Source: Popul Environ. 2025 Sep 25;47(4):34. doi: 10.1007/s11111-025-00506-6 (PMC12464131; doi:10.1007/s11111-025-00506-6)
Supplement: Supplementary file 5 — (DOCX 18.8 KB) [file 11111_2025_506_MOESM5_ESM.docx]

# Appendix S5: Interview and Focus Group Questions

**Interview questions**

| Question | Prompt |
| --- | --- |
| 1. How many children do you have? |  |
| 1. How old are you, and what age(s) are your children? |  |
| 1. How is your home heated? | Gas boiler, electric storage heaters, etc. |
| 1. Please tell me about your home heating patterns. Has this changed after having a child? | Timer, thermostat settings |
| 1. Thinking now about activities you do to conserve energy at home, do you often turn the lights off when you leave the room? | Do you encourage your children to do the same? |
| 1. Turning to transport, tell me about a typical week of travel for you. Which are the main modes of transport you use? | Has this changed since having children? |
| 1. How many times a year would you say you fly? Has this changed since having children? Do you usually, or have you ever, offset the emissions from your flights? | Do you ever think about the environmental impacts of flying? |
| 1. Please tell me about your family’s diet. Do you eat fish and/or meat at home? | Do you ever consider the environmental consequences of the food you and your family eat? |
| 1. Do you use disposable or reusable nappies? |  |
| 1. When I say climate change, what three words come to mind? |  |
| 1. How knowledgeable are you about climate change and other environmental issues? | On a scale of 1-5, how would you rate your knowledge? |
| 1. How serious of a threat do you think climate change is to you and your family? |  |
| 1. Do you think the UK government is doing enough to tackle climate change? |  |
| 1. Do you think it is important to foster a sense of love for the environment in your children? |  |
| 1. What is the biggest change that has occurred in your life after having children? | Have you moved house, bought a car, changed heating system, got a new job? |
| 1. Whose responsibility is it to teach your children about climate change? | [for parents of older children] have you ever spoken with your children about climate? How did you approach this? What did you discuss? What prompted it? |
| 1. Do you think your perception of climate change has changed after having children? | In what ways? |
| 1. Have you and your partner ever had any discussions surrounding having another child during the climate crisis? |  |
